# Efferocytosis‐Driven Reduction of Neuroinflammation and Amyloid Beta Burden via Novel GAS6 Fusion Protein, GAIA: A Promising Therapeutic Approach in Alzheimer's Disease

**DOI:** 10.1002/alz70859_101799

**Published:** 2025-12-25

**Authors:** Jin Kyung Lee, Juho Lee, Jaemin Shin, Eun Jung Lee, Sung Wook Kim, Seong Beom An, Heeju Han, Gyunam Jang, Bokyung Kim, Sanghoon Park

**Affiliations:** ^1^ ILLIMIS THERAPEUTICS, Seoul, Gangnam‐gu Korea, Republic of (South)

## Abstract

**Background:**

Amyloid‐beta (Aβ) accumulation is a hallmark of Alzheimer's disease (AD), and reducing Aβ burden is a key therapeutic strategy. Recent FDA‐approved anti‐Aβ antibodies, such as Lecanemab and Donanemab, have demonstrated significant reductions in Aβ burden and deceleration of cognitive decline. However, these therapies are associated with adverse events, including antibody‐induced neuroinflammation and amyloid‐related imaging abnormalities (ARIA). The GAIA platform leverages TAM receptors—Tyro3, Axl and MerTK—to facilitate efferocytosis‐driven Aβ clearance without triggering inflammatory responses, addressing limitations of current anti‐Aβ immunotherapies. This study evaluates the pharmacokinetic properties and therapeutic efficacy of GAIA‐Aβ.

**Method:**

GAIA‐Aβ was engineered with dual functional domains, a GAS6 domain for TAM receptor binding and Aβ‐targeting moiety. Specific binding to oligomeric Aβ (oAβ) and TAM receptors was confirmed using ELISA. TAM receptor‐driven phagocytosis and oAβ clearance were evaluated using HMC3, human microglial cell line. Additionally, anti‐inflammatory responses were assessed in induced pluripotent stem cell (iPSC)‐derived monocytes. Pharmacokinetic properties of GAIA‐Aβ were analyzed to determine serum exposure and brain‐to‐serum ratio. To evaluate the in vivo efficacy, GAIA‐Aβ was intravenously administered to 5xFAD mice once weekly (QW) for 8 weeks, and Aβ plaque burden and glial phagocytic activity were assessed using immunohistochemistry.

**Result:**

GAIA‐Aβ exhibited specific binding to oAβ and activated TAM receptors in a dose‐dependent manner. Phagocytosis assays demonstrated effective clearance of oAβ while reducing inflammatory cytokines, indicating successful efferocytosis‐mediated activity. Pharmacokinetic analysis revealed that GAIA‐Aβ possesses properties comparable to conventional monoclonal antibodies. In 5xFAD mice, GAIA‐Aβ treatment led to significant reductions in Aβ plaques and enhanced glial‐mediated clearance, particularly via astrocyte engagement.

**Conclusion:**

GAIA‐Aβ effectively reduces Aβ burden while mitigating neuroinflammation, presenting a favorable safety profile compared to existing anti‐Aβ antibodies. These findings highlight the potential of GAIA‐Aβ as an improved therapeutic approach for Alzheimer's disease, addressing the limitations of current anti‐Aβ immunotherapies.

